# Supplementary material for: Transcriptomic profiling of mTOR and ryanodine receptor signaling molecules in developing zebrafish in the absence and presence of PCB 95
Source: PeerJ. 2017 Nov 29;5:e4106. doi: 10.7717/peerj.4106 (PMC5712209; doi:10.7717/peerj.4106)
Supplement: Supplemental Information 1 [file peerj-05-4106-s001.docx]

# SUPPLEMENTAL MATERIAL

**Transcriptomic profiling of mTOR and ryanodine receptor signaling molecules in developing zebrafish in the absence and presence of PCB 95**

Daniel F. Frank^1,2^, Galen W. Miller^3^, Richard E. Connon^1^, Juergen Geist^2^, and Pamela J. Lein^3^*

^1^ Department of Anatomy, Physiology and Cell Biology, University of California, Davis, School of Veterinary Medicine, Davis, CA 95616, USA

^2^ Aquatic Systems Biology, Department of Ecology and Ecosystem Management, Technical University Munich, Mühlenweg 22, D-85354 Freising, Germany

^3^ Department of Molecular Biosciences, University of California, Davis, School of Veterinary Medicine, Davis, CA 95616, USA

* Corresponding author


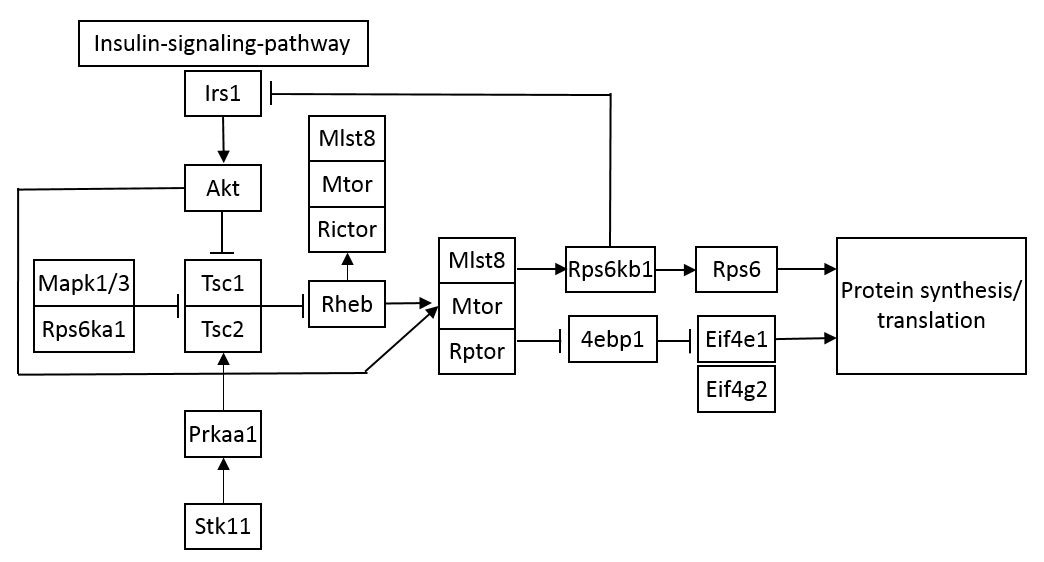


**Figure S1: Schematic of mTOR signaling.** Schematic illustrating the functional relationship of the mTOR signaling molecules analyzed in this study. Eif4g2 is not directly affected by upstream members of the signaling pathway, but is part of a complex formed with eif4e1.


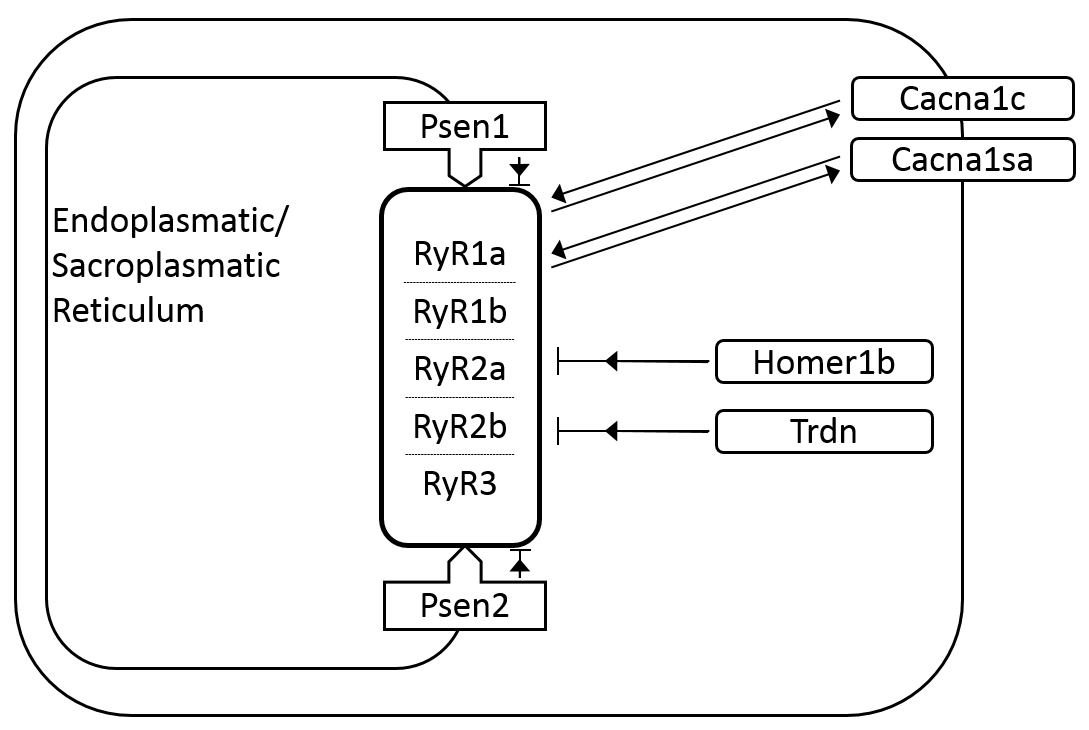


**Figure S2: Ryanodine receptor (RyR) signaling molecules in the zebrafish.** Schematic listing all the zebrafish ryanodine receptors (RyR) paralogs and regulatory proteins investigated in this study. Arrows highlight regulatory proteins that stabilize the RyR in its open configuration, which increases release of Ca^2+^ from internal stores; whereas blunt ends identify protein interactions that inhibit RyR activity.

**Figure S3. Electrophoresis run file summary.** Bioanalyzer output corresponding to Table S3 RIN scores. Illustrated are all 24 hpf, 72 hpf and 120 hpf samples, with the exception of one 72hpf sample (72-3).

**
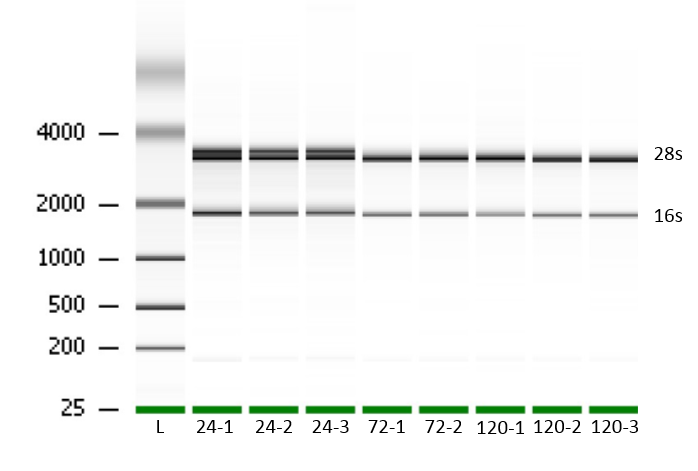
**

**Table S1:** **Genes from mTOR signaling selected for transcriptomic analysis.** Transcripts indicated in bold font were quantified in the PCB 95 exposure studies.

| **Gene name** | **Gene code** | **Primer (5'->3')** | **Accession #** | **Efficiency %** | **Amplicon**  **lengths** | **Exon** | **Chromo**  **some** |
| --- | --- | --- | --- | --- | --- | --- | --- |
| **Target genes**  Insulin receptor substrate 1 | *irs1* | **F:** GCTCAGTGCCTATGCCAGTA  **R:** AAGCAGCGGCGGATTTTTAC | XM_682610 | 91.2 | 99 | 1-2 | 15 |
| V-akt murine thymoma viral oncogene homolog 1 | *akt1* | **F:** TAAGGAGCGACCGCAAGATG  **R:** TGCAGGCAGCGTATGATGAA | NM_001281801 | 91.9 | 123 | 1-4 | 17 |
| Tuberous sclerosis 1a | *tsc1a* | **F:** TCACGACACCCATGGGAAAG  **R:** TGCAGGCACAAGACCTTTCAA | NM_200052 | 96.9 | 88 | 14-16 | 5 |
| Tuberous sclerosis 2 | *tsc2* | **F:** AGTATGACGTGGCTGGTTGG  **R:** TCTTTGGTCTGTCGGGTGTG | XM_009294973 | 91.3 | 155 | 24-25 | 1 |
| Ras homolog enriched in brain | *rheb* | **F:** TTGGACATGGTGGGGAAAGT  **R:** TTCACAGCTGATCACTCGCT | NM_200729 | 95.9 | 87 | 1-3 | 24 |
| mTOR associated protein | ***mlst8*** | **F:** ACATGCTCTGCTGACCAGAC  **R:** CACAATCCCACATCCAGCCT | NM_199877 | 101.5 | 121 | 7 | 12 |
| Mechanistic target of Rapamycin | ***mtor*** | **F:** ATGGTCACTGGCCTGAAGTG  **R:** GTGCACGTGGCGTATCAATC | NM_001077211 | 102.1 | 117 | 41-42 | 8 |
| Rptor regulatory associated protein of MTOR | ***rptor*** | **F:** TTCATCAAGCTGGCGGATCTC  **R:** CATCTTCCTGGTGCGTGGA | XM_005157354 | 96.5 | 170 | 23-24 | 6 |
| Rptor independent companion of mTOR, complex 2a | ***rictora*** | **F:** ATCTGATCCGTGACAGCAGC  **R:** CCAATCGGAGGGCTTGAGTT | XM_009301197 | 102.2 | 123 | 5-6 | 5 |
| eukaryotic translation initiation factor 4E binding protein 1 | ***eif4ebp1*** | **F:** ACATGGGGGACGTTTTCACA  **R:** GGAGTTGGATTTCCCCCACA | NM_199645 | 102.4 | 149 | 4 | 21 |
| p70 ribosomal S6 kinase a (*p70s 6ka*) | rps6kb1a | **F:** ACAGCCCTGATGACACGAAG  **R:** TTCTTGGGCTTCCCAGAACC | XM_685634 | 101.8 | 153 | 13-14 | 10 |
| p70 ribosomal S6 kinase b (*p70s 6kb*) | rps6kb1b | **F:** TGACTGATTTCGGGCTGTGT  **R:** CGATTGTGTCCGCTCCTCAT | NM_213076 | 94.9 | 115 | 8-9 | 15 |
| Eukaryotic translation initiation factor 4e1a | *eif4e1a* | **F:** TCGTATTGCAGCTTGAGAATGT  **R:** TGACATGAGGAATGTGGAACA | NM_131733 | 108.7 | 97 | 8 | 14 |
| Eukaryotic translation initiation factor 4e1b | *eif4e1b* | **F:** GGTCAAAGAGTCATCATCTTGTATTG  **R:** TCACAAAGTCTTGCATATTTCAAGTT | NM_131454 | 97.0 | 95 | 7 | 5 |
| Eukaryotic translation initiation factor 4e1c | *eif4e1c* | **F:** TTCGGAGCCGCGAGGA  **R:** AGATGAGACGCAGGTTTTCTGT | NM_001017851 | 91.9 | 170 | 1-3 | 13 |
| Eukaryotic translation initiation factor 4, gamma 2a | *eif4g2a* | **F:** GAAAGAAGACATTACCCAGGAG  **R:** CAGCCACATCAGCCACTG | NM_001014289 | 100.2 | 76 | 21-22 | 7 |
| Eukaryotic translation initiation factor 4, gamma 2b | *eif4g2b* | **F:** GGTGCTTCTCGTTTCAGTGC  **R:** TGCCGACAGTCTTGGGATA | NM_001013443 | 101.5 | 79 | 2-3 | 18 |
| Ribosomal protein S6 kinase 1 (s6) | ***rps6*** | **F:** TCTGAGCCCTTACGCTTTCG  **R:** TCTGTTGGGTGGGGAGATGAT | EF373681 | 100.9 | 99 | 1-2 | 1 |
| AMP-activated, alpha 1 catalytic subunit (*ampk*) | prkaa1 | **F:** AGAGCAGGCCGAATGACATC  **R:** GACTGGGTTCTTCCTTCGCA | NM_001110286 | 90.6 | 113 | 8-9 | 5 |
| serine/threonine kinase 11 (*lkb1*) | stk11 | **F:** CACATGGATGCAGTTCCCGTA  **R:** ACTGGAGATCCGGACATCTCT | NM_001017839 | 93.3 | 70 | 2 | 2 |
| Mitogen-activated protein kinase 3 (*erk1*) | mapk3 | **F:** TGACCGACTCCTCTTCGACT  **R:** CGATTCCGCCATCGTTTCTG | NM_201507 | 102.7 | 158 | 1 | 3 |
| Mitogen-activated protein kinase 1 (*erk2*) | mapk1 | **F:** GCTTTTCGCCGTGTTTCACT  **R:** CTGAAGCTGCCTTCACAAGC | NM_182888 | 90.1 | 92 | 1 | 5 |
| Ribosomal protein S6 kinase a, polypeptide 1 (*rsk*) | rps6ka1 | **F:** AATGCAGTTGTGGACCGGAT  **R:** TCAGCCAATCAGCTCCCTTG | NM_001077775 | 92.4 | 166 | 8 | 17 |

**Table S2:** **Genes from RyR signaling selected for transcriptomic analysis and in situ analysis.** Transcripts indicated in bold font were quantified in the PCB 95 exposure studies.

| **Gene name** | **Gene code** | **Primer (5'->3')** | **Accession #** | **Efficiency %** | **Amplicon**  **lengths** | **Exon** | **Chromo some** |
| --- | --- | --- | --- | --- | --- | --- | --- |
| **Reference genes** |  |  |  |  |  |  |  |
| Beta-actin | *actb2* | **F:** AAGCAGGAGTACGATGAGTC  **R:** TGGAGTCCTCAGATGCATTG | NM_181601 | 101.7 | 238 | 6 | 3 |
| Elongation factor 1 alpha | *eef1a1* | **F:** GATGCACCACGAGTCTCTGA  **R:** TGATGACCTGAGCGTTGAAG | NM_131263 | 99.1 | 158 | 6 | 19 |
| **Target genes** |  |  |  |  |  |  |  |
| Ryanodine receptor 1a | ***ryr1a*** | **F:** TCCTGCTACCGAATCATGTG  **R:** GCCTCTCTGCATGTGGAGTT | XM_009305502 | 97.0 | 67 | 67-68 | 10 |
| Ryanodine receptor 1b | ***ryr1b*** | **F:** AAGAAATCGGGCATCTGGCA  **R:** TGAGTTGTTCGGAGGTGACG | NM_001102571 | 90.6 | 157 | 55 | 18 |
| Ryanodine receptor 2a | ***ryr2a*** | **F:** AGGACTCAAGCCAAATCGAG  **R:** TCACGACCATGTCCTTCTGA | XM_009300703 | 107.0 | 63 | 6-7 | 12 |
| Ryanodine receptor 2b | ***ryr2b*** | **F:** TCCTTTAGTCATTTTTAAGCGAGA  **R:** GTCCATCGAACTCCAGTTTACG | XM_017351708 | 97.9 | 59 | 93 | 17 |
| Ryanodine receptor 3 | ***ryr3*** | **F:** TCTTCGCTGCTCATTTGTTG  **R:** AGACAGGATGGTCCTCAAGGT | AB355791 | 103.9 | 62 | 97 | 20 |
| Homer homolog 1b | *homer1b* | **F:** GGAGGGAAAAACAACAGCAA  **R:** GGGGTCTATCTGGAAGACGTG | XM_005157295 | 91.7 | 75 | 1-2 | 5 |
| calcium channel, voltage-dependent,  L type, alpha 1C subunit | *cacna1c* | **F:** ATATCTTCAGGCGGTCTGGTG  **R:** GACTGTGGGAAGGAGACGTG | NM_131900 | 100.3 | 85 | 42-43 | 4 |
| calcium channel, voltage-dependent,  L type, alpha 1S subunit, a | *cacna1sa* | **F:** GATAGCAGAGCGGACAGGAC  **R:** TGCATGCTGGGAAATGTGGT | NM_001146150 | 97.8 | 115 | 42-23 | 22 |
| Presenilin 1 | *psen1* | **F:** TTGACATCGGGAGCATCGTT  **R:** TACAAAACACTCGCGTTTCCTT | NM_131024 | 90.6 | 180 | 11 | 17 |
| Presenilin 2 | *psen2* | **F:** TCAAATACGGCGCGAAACAC  **R:** GTCCGCTCTTCTCGGTGTAG | NM_131514 | 91.4 | 111 | 3 | 1 |
| Triadin | *trdn* | **F:** AAGGAAGCAGGCAGCCTTAG  **R:** TTCTTCCGGCGCTACCAAAT | XM_009294909 | 94.8 | 135 | 3-4 | 20 |
| Wingless-type 2ba | ***wnt2ba*** | **F:** AAGTACAACGCTGCTGTGGA  **R:** GAACCTGCTGATCGGTCCAT | NM_182876 | 92.2 | 149 | 4-5 | 6 |
| Wingless-type 2bb | *wnt2bb* | **F:** TTCGTCATCACCGCTGGAAT  **R:** TCCACGTTTCTGTGGGTCAC | NM_001044344 | 92.3 | 191 | 2-3 | 8 |
|  |  |  |  |  |  |  |  |
| **Probe for *in situ* hybridization** | **Primer (5'->3')** | | | **Accession #** | **Amplicon**  **lengths** | **Exon** | **Chromo some** |
| Ryanodine receptor 3 | **F:** CCTTGCTCTGCGTCTTTGGA  **R: CATTAACCCTCACTAAAGGGAA**GCCACAACGGTGTAGAGGTAA | | | AB355791 | 601 | 92-97 | 20 |

| **Sample** | **RIN** |
| --- | --- |
| 24-1 | 8.50 |
| 24-2 | 8.70 |
| 24-3 | 8.50 |
| 72-1 | 10 |
| 72-2 | 9.90 |
| 120-1 | 9.80 |
| 120-2 | 10 |
| 120-3 | 10 |

**Table S3: Bioanalyzer results.** RNA integrity ratio (RIN) scores above 8 were considered good quality.

**CT-values for the ontogenetic profile of gene expression**

|  | **24 - 1** | **24-2** | **24 - 3** | **72-1** | **72-2** | **72-3** | **120-1** | **120-2** | **120-3** |
| --- | --- | --- | --- | --- | --- | --- | --- | --- | --- |
| **actb2** | 13.72664 | 13.84284 | 13.78229 | 14.67406 | 13.77039 | 14.8818 | 14.31989 | 13.54781 | 13.70186 |
| **eef1a1** | 11.78943 | 11.81506 | 11.69878 | 12.98444 | 12.46467 | 11.35629 | 12.61911 | 12.0681 | 11.93378 |
| **ryr1a** | 22.51039 | 21.05069 | 21.48023 | 21.58823 | 21.64727 | 21.88504 | 21.45028 | 21.69171 | 21.66002 |
| **ryr 1b** | 19.4976 | 19.3478 | 19.20753 | 20.79686 | 19.69899 | 20.37269 | 19.44418 | 19.42794 | 18.52753 |
| **ryr2a** | 25.18421 | 24.68323 | 24.31422 | 22.53933 | 22.18175 | 23.0569 | 22.0339 | 21.9991 | 22.0031 |
| **ryr2b** | 25.73279 | 24.63189 | 25.08385 | 21.81379 | 25.55721 | 26.7313 | 24.09973 | 25.8489 | 25.92664 |
| **ryr3** | 25.51072 | 23.13439 | 23.05046 | 22.32243 | 22.01386 | 19.34913 | 19.93659 | 19.33287 | 19.35913 |
| **homer1b** | 23.39763 | 22.30726 | 21.71593 | 21.74192 | 20.30429 | 20.70009 | 20.69878 | 20.14123 | 19.84086 |
| **cacnac1c** | 24.78385 | 23.97956 | 24.01378 | 23.06017 | 21.74773 | 22.61156 | 21.63266 | 21.78646 | 21.87678 |
| **cacnac1sa** | 23.50158 | 22.76859 | 22.05743 | 21.57031 | 21.20989 | 21.26385 | 22.2285 | 22.05453 | 21.94775 |
| **psen1** | 23.61253 | 22.62173 | 23.63443 | 22.34947 | 22.86818 | 22.58382 | 23.55399 | 23.33145 | 23.89983 |
| **psen2** | 18.94463 | 18.71563 | 18.84544 | 18.8242 | 18.6667 | 18.78965 | 18.84897 | 19.03766 | 18.84691 |
| **trdn** | 18.55669 | 17.87031 | 17.85886 | 18.69736 | 17.50456 | 18.02566 | 17.9914 | 17.94229 | 17.99253 |
| **wnt2ba** | 25.60267 | 24.96852 | 24.9272 | 24.30795 | 24.27728 | 24.17033 | 23.88881 | 22.93738 | 22.01935 |
| **wnt2bb** | 22.44901 | 22.01574 | 22.05962 | 23.53335 | 22.68696 | 23.10041 | 23.14674 | 23.6249 | 23.14696 |
| **irs1** | 20.75614 | 20.96163 | 20.96935 | 22.74458 | 21.19034 | 20.06044 | 21.64484 | 20.77017 | 21.29776 |
| **akt1** | 21.15625 | 20.93731 | 20.30514 | 22.50157 | 21.51923 | 22.1004 | 22.13722 | 20.85499 | 21.78861 |
| **tsc1a** | 23.32317 | 22.80225 | 23.02401 | 23.48926 | 22.09123 | 22.63692 | 22.58711 | 22.38968 | 21.35034 |
| **tsc2** | 21.30718 | 20.49048 | 20.61872 | 21.28084 | 19.60084 | 22.15566 | 20.6867 | 19.03718 | 20.31987 |
| **rheb** | 21.68246 | 23.06439 | 23.09804 | 21.71885 | 21.65687 | 21.59007 | 21.24458 | 21.14368 | 21.09227 |
| **mlst8** | 21.17114 | 21.69932 | 21.86005 | 21.79732 | 21.73501 | 22.36708 | 22.31334 | 22.25386 | 21.91824 |
| **mtor** | 21.79581 | 20.83639 | 20.38849 | 22.86695 | 21.16189 | 21.40873 | 21.60065 | 21.55669 | 21.49572 |
| **rptor** | 23.08695 | 22.76144 | 23.80133 | 24.95673 | 23.86857 | 24.38265 | 23.93254 | 24.07232 | 24.07001 |
| **rictora** | 19.45717 | 20.51325 | 19.45178 | 21.44015 | 20.39925 | 22.04105 | 19.54233 | 18.68876 | 18.38026 |
| **eif4ebp1** | 20.33786 | 20.26522 | 20.53935 | 21.22676 | 20.84071 | 21.12321 | 21.15043 | 21.43518 | 20.14032 |
| **rps6kb1a** | 22.20109 | 21.83982 | 21.89656 | 21.97604 | 21.21313 | 21.49565 | 21.42315 | 22.14746 | 21.50527 |
| **rps6kb1b** | 18.65267 | 19.37857 | 18.65882 | 19.5104 | 19.54416 | 20.01148 | 20.19964 | 20.44261 | 19.82265 |
| **eif4e1a** | 21.45664 | 22.59849 | 23.10565 | 21.25996 | 23.13968 | 24.59979 | 23.76182 | 24.8119 | 24.50423 |
| **eif4e1b** | 28.48961 | 27.99437 | 28.31032 | 29.68151 | 28.97203 | 30.43099 | 28.79792 | 29.31764 | 28.39821 |
| **eif4e1c** | 19.13912 | 17.72278 | 18.07906 | 20.50353 | 17.85354 | 19.21854 | 19.35947 | 19.16192 | 20.70317 |
| **eif4g2a** | 20.74799 | 20.39486 | 19.74132 | 22.03249 | 20.60574 | 20.97386 | 22.31593 | 21.65899 | 20.26669 |
| **eif4g2b** | 18.18206 | 17.17442 | 17.46095 | 19.42931 | 17.59898 | 18.75553 | 17.83813 | 17.77436 | 17.78073 |
| **rps6** | 21.99504 | 23.1802 | 23.53114 | 22.09121 | 23.99747 | 26.11466 | 23.07566 | 25.19126 | 24.68419 |
| **prkaa1** | 21.75832 | 21.40698 | 20.92536 | 21.94039 | 20.73427 | 21.43921 | 21.75981 | 21.79776 | 20.1247 |
| **stk11** | 21.44071 | 21.29941 | 20.91631 | 21.59753 | 21.03212 | 21.4459 | 21.25372 | 20.88585 | 21.49167 |
| **mapk3** | 17.79698 | 17.89578 | 17.95972 | 18.81617 | 17.87005 | 18.32353 | 18.79679 | 17.44108 | 17.98195 |
| **mapk1** | 21.24384 | 20.8862 | 20.4871 | 20.96638 | 19.33732 | 20.32095 | 20.31909 | 20.22053 | 19.00896 |
| **rps6ka1** | 22.22298 | 21.72043 | 22.07399 | 20.08908 | 20.01569 | 20.05586 | 20.19429 | 20.37231 | 20.22468 |

**CT-values for the PCB95 exposure study:**

|  | **actb2** | **eef1a1** | **mtor** | **rptor** | **rictora** | **mlst8** | **rps6** |
| --- | --- | --- | --- | --- | --- | --- | --- |
| **72-1-dmso** | 14.68519 | 13.34043 | 21.47908 | 23.49884 | 21.22138 | 21.54427 | 23.10684 |
| **72-1-0.1** | 14.98128 | 14.22077 | 21.52462 | 24.1039 | 21.53748 | 21.95176 | 23.54168 |
| **72-1-0.3** | 14.79923 | 13.45915 | 21.49316 | 23.69937 | 21.80255 | 21.97251 | 23.08783 |
| **72-1-1.0** | 14.86704 | 13.90054 | 21.83134 | 24.22452 | 22.2325 | 21.84172 | 23.45149 |
| **72-1-3.0** | 15.08526 | 13.9519 | 21.66972 | 24.52133 | 22.2266 | 22.11789 | 23.23497 |
| **72-1-10.0** | 15.10167 | 14.36444 | 22.22737 | 25.79081 | 22.17169 | 21.66137 | 22.93566 |
| **72-2-dmso** | 14.8466 | 13.76666 | 21.56612 | 23.97287 | 21.80739 | 21.92094 | 23.26845 |
| **72-2-0.1** | 15.01117 | 13.94358 | 21.72335 | 23.97296 | 21.89125 | 21.53627 | 23.09022 |
| **72-2-0.3** | 14.40841 | 13.92387 | 21.59 | 24.16887 | 21.30178 | 21.72232 | 23.42518 |
| **72-2-1.0** | 15.05858 | 14.01102 | 21.71728 | 24.32576 | 22.0416 | 22.14021 | 23.44266 |
| **72-2-3.0** | 14.96282 | 14.00707 | 22.01779 | 24.86734 | 22.13723 | 21.91939 | 23.37404 |
| **72-2-10.0** | 14.8702 | 14.10744 | 21.84276 | 25.30812 | 21.80993 | 21.55936 | 23.11228 |
| **72-3-dmso** | 13.35047 | 13.29042 | 21.27483 | 23.80408 | 21.35091 | 21.76362 | 23.27171 |
| **72-3-0.1** | 13.59782 | 13.73892 | 20.89305 | 23.22045 | 21.15394 | 21.18453 | 22.87158 |
| **72-3-0.3** | 14.71382 | 13.69286 | 21.43231 | 23.65576 | 21.80541 | 21.75507 | 23.39066 |
| **72-3-1.0** | 14.84718 | 13.22551 | 21.4965 | 23.88741 | 21.34238 | 21.73286 | 23.34588 |
| **72-3-3.0** | 14.93224 | 13.9176 | 21.49784 | 24.36126 | 21.51039 | 21.36623 | 22.66808 |
| **72-3-10.0** | 14.50926 | 12.98636 | 21.04477 | 24.5075 | 21.09175 | 21.18884 | 22.6378 |
| **120-1-dmso** | 14.62455 | 13.43891 | 21.84645 | 23.89682 | 21.21622 | 21.87665 | 23.50016 |
| **120-1-0.1** | 14.74216 | 13.40048 | 21.62999 | 23.88057 | 21.182 | 21.53005 | 22.91563 |
| **120-1-0.3** | 14.25444 | 13.5683 | 21.73482 | 24.20011 | 21.61442 | 21.75118 | 22.94169 |
| **120-1-1.0** | 13.56497 | 12.82785 | 21.23527 | 24.28485 | 21.25745 | 21.8066 | 22.76389 |
| **120-1-3.0** | 14.55749 | 12.88863 | 21.33303 | 24.90198 | 21.24337 | 21.99675 | 23.04947 |
| **120-1-10.0** | 15.1301 | 13.92025 | 21.98581 | 25.51365 | 21.74669 | 22.38849 | 23.0653 |
| **120-2-dmso** | 14.98583 | 13.19768 | 21.90735 | 24.04222 | 22.07656 | 22.25399 | 23.66646 |
| **120-2-0.1** | 13.95884 | 12.63348 | 21.9237 | 23.77004 | 21.41178 | 21.73003 | 23.49548 |
| **120-2-0.3** | 14.88289 | 13.61427 | 21.86662 | 24.22769 | 21.55571 | 21.90129 | 23.43539 |
| **120-2-1.0** | 14.7598 | 13.5797 | 21.91445 | 24.55876 | 21.47965 | 21.83218 | 23.54177 |
| **120-2-3.0** | 15.44692 | 14.02251 | 22.2112 | 25.27382 | 22.01824 | 22.14386 | 23.08174 |
| **120-2-10.0** | 14.95425 | 14.04371 | 22.27522 | 25.85607 | 21.9371 | 22.44353 | 24.08389 |
| **120-3-dmso** | 14.52348 | 12.92704 | 21.39418 | 23.43069 | 21.22625 | 21.58492 | 23.1202 |
| **120-3-0.1** | 14.151 | 13.90857 | 21.88882 | 24.4729 | 21.39214 | 22.06254 | 23.70301 |
| **120-3-0.3** | 14.40291 | 12.72141 | 21.43071 | 23.83032 | 21.29306 | 21.57149 | 23.26682 |
| **120-3-1.0** | 14.81479 | 13.79213 | 22.01532 | 24.7902 | 22.15145 | 22.24179 | 23.45776 |
| **120-3-3.0** | 15.21333 | 14.13882 | 22.30159 | 25.55404 | 22.0247 | 22.03243 | 23.32257 |
| **120-3-10.0** | 14.7008 | 13.49178 | 21.78264 | 25.33345 | 21.19056 | 21.61818 | 23.17982 |

|  | **eif4ebp1** | **ryr1a** | **ryr 1b** | **ryr2a** | **ryr2b** | **ryr3** | **wnt2ba** |
| --- | --- | --- | --- | --- | --- | --- | --- |
| **72-1-dmso** | 20.66015 | 21.69773 | 20.19932 | 22.30695 | 24.81088 | 20.50408 | 23.01835 |
| **72-1-0.1** | 20.82525 | 22.2189 | 20.62988 | 22.72796 | 25.15827 | 20.96602 | 23.52431 |
| **72-1-0.3** | 20.97774 | 21.85977 | 20.45428 | 22.56317 | 24.74801 | 20.66306 | 23.00072 |
| **72-1-1.0** | 20.86102 | 22.06641 | 20.78848 | 22.98522 | 25.21059 | 20.81267 | 23.53021 |
| **72-1-3.0** | 20.87895 | 22.05544 | 20.54416 | 22.63654 | 24.55034 | 20.86942 | 22.96894 |
| **72-1-10.0** | 21.13125 | 22.06765 | 20.82214 | 22.66275 | 23.91958 | 21.1711 | 22.42716 |
| **72-2-dmso** | 21.25023 | 21.96913 | 20.64156 | 22.671 | 25.02934 | 20.62955 | 23.34655 |
| **72-2-0.1** | 20.94125 | 21.84458 | 20.53378 | 22.92567 | 24.66081 | 20.78065 | 23.19858 |
| **72-2-0.3** | 20.86431 | 22.17745 | 20.71266 | 22.76683 | 25.13466 | 20.87597 | 23.36054 |
| **72-2-1.0** | 21.06222 | 22.08502 | 20.56841 | 22.70431 | 24.97572 | 20.93803 | 23.5641 |
| **72-2-3.0** | 20.91841 | 22.06619 | 20.90415 | 22.8005 | 24.83784 | 20.95613 | 23.34849 |
| **72-2-10.0** | 20.76094 | 21.93378 | 20.68048 | 22.74902 | 24.46411 | 20.91498 | 22.96442 |
| **72-3-dmso** | 20.53361 | 21.59452 | 19.93883 | 22.4109 | 24.68173 | 20.61698 | 22.97388 |
| **72-3-0.1** | 20.30644 | 21.43117 | 19.81826 | 22.02061 | 24.49696 | 20.25707 | 22.91836 |
| **72-3-0.3** | 20.8102 | 21.7551 | 20.22651 | 22.46152 | 25.17404 | 20.4037 | 23.34087 |
| **72-3-1.0** | 20.90088 | 21.91696 | 20.28874 | 22.65494 | 24.73076 | 20.45826 | 23.14229 |
| **72-3-3.0** | 20.61425 | 21.61337 | 20.14891 | 22.33578 | 24.15008 | 20.71102 | 22.56212 |
| **72-3-10.0** | 20.40542 | 21.17654 | 19.76212 | 22.1064 | 23.93717 | 20.1188 | 22.34961 |
| **120-1-dmso** | 20.90247 | 22.30588 | 20.21812 | 22.57459 | 25.17103 | 20.02108 | 23.40214 |
| **120-1-0.1** | 20.78569 | 21.76109 | 19.90602 | 22.2901 | 24.84759 | 19.93727 | 22.94018 |
| **120-1-0.3** | 20.86502 | 22.01787 | 20.04695 | 22.45709 | 24.66563 | 20.16909 | 23.00896 |
| **120-1-1.0** | 20.38754 | 21.5804 | 19.56055 | 22.13743 | 24.6136 | 19.5923 | 22.65974 |
| **120-1-3.0** | 20.69693 | 21.77081 | 19.89614 | 22.21744 | 24.79831 | 19.77448 | 23.00591 |
| **120-1-10.0** | 21.17063 | 22.03223 | 20.59187 | 22.54759 | 24.59109 | 20.75662 | 22.96616 |
| **120-2-dmso** | 21.09998 | 22.35442 | 20.53305 | 22.26226 | 25.11628 | 20.32128 | 23.47201 |
| **120-2-0.1** | 20.70559 | 22.28285 | 20.39975 | 22.64434 | 24.96739 | 20.43465 | 23.38632 |
| **120-2-0.3** | 20.95442 | 22.44162 | 20.59395 | 22.28493 | 24.84576 | 20.42891 | 23.35277 |
| **120-2-1.0** | 20.97036 | 22.37042 | 20.5085 | 22.62453 | 25.22594 | 20.35144 | 23.43907 |
| **120-2-3.0** | 21.14825 | 22.33062 | 20.69029 | 22.34038 | 24.26096 | 20.67543 | 22.8081 |
| **120-2-10.0** | 21.03551 | 22.5301 | 21.11663 | 23.11973 | 25.49439 | 21.11901 | 23.79692 |
| **120-3-dmso** | 20.70459 | 21.912 | 20.18827 | 21.95744 | 24.75588 | 19.92775 | 22.95448 |
| **120-3-0.1** | 21.2562 | 22.50382 | 20.55401 | 22.57015 | 25.00438 | 20.48607 | 23.66652 |
| **120-3-0.3** | 20.7042 | 22.0136 | 20.27387 | 22.20374 | 24.89158 | 19.97381 | 23.14522 |
| **120-3-1.0** | 21.05866 | 22.54522 | 20.82768 | 22.73667 | 24.86723 | 20.78724 | 23.40476 |
| **120-3-3.0** | 21.17576 | 22.51461 | 20.99032 | 22.65776 | 24.67053 | 20.86155 | 23.10949 |
| **120-3-10.0** | 20.70863 | 21.96247 | 20.40139 | 22.25757 | 24.70481 | 20.67187 | 23.03124 |

**R-code:**

library(stats)

library(multcomp)

library(lme4)

library(nlme)

library(pgirmess)

library(coin)

library(PMCMR)

setwd('')

house_qpcr<-read.csv('', header=T)

house_qpcr

house_qpcr$hpf<-factor(house_qpcr$hpf)

house_qpcr$rep<-factor(house_qpcr$rep)

str(house_qpcr)

**Test for Normality**:

shapiro.test(house_qpcr$genecode)

bartlett.test(genecode~hpf, data= house_qpcr)

significance test for nonparametric data (not normally distributed), followed by a post hoc test (comparing the single treatments)

Kruskal Wallis

kruskal.test(genecode~hpf, data= house_qpcr)

kruskalmc(house_qpcr$genecode, house_qpcr$hpf)

**Dunn's test:**

attach(house_qpcr)

posthoc.kruskal.nemenyi.test(x=genecode, g=treatment, method="Tukey")

detach(house_qpcr)

##significance test for parametric data ( normally distributed), followed by a post hoc test (comparing the single treatments)

gene codealm<-lm(p70s6kb~hpf, data=house_qpcr)

summary(gene codealm)

par(mfrow=c(2,2))

plot(gene codealm)

gene codealmtukey<-glht(gene codealm,linfct=mcp(hpf="Tukey"))

summary(gene codealmtukey)

**Graphs**:

library(ggplot2)

library(bear)

library(Rmisc)

##Dataset including the Handling Control and the Tank Control

setwd('')

graph<-read.csv('', header=T)

graph

graph$hpf<-factor(graph$hpf)

graph$gene<-factor(graph$gene)

#graph$conc<-factor(graph$conc)

str(graph)

head(graph)

#summarySE provides the standard deviation, standard error of the mean, and a (default 95%) confidence interval

graphc<-summarySE(graph, measurevar="CT", groupvars=c("hpf","gene"))

graphc

pd <- position_dodge(0.1)

ggplot(graphc, aes(x=hpf, y=CT, group=gene, colour=gene, linetype=gene)) +

geom_errorbar(aes(ymin=CT-se, ymax=CT+se), colour="black", width=.3, position=pd, linetype=1) +

geom_line( size=1.2, position=pd) +

geom_point(position=pd, size=3, shape=21, fill="white") +

expand_limits(y=5:-3) +

theme_bw(base_size = 20) +

theme(panel.grid.major = element_line(color = "black", linetype = "dotted"), legend.justification=c(0,1), legend.position = c(0,1), legend.title=element_blank()) +

labs(x = "hours post fertilization",y="Log2(-\u0394\u0394CT ")
